# Narrative Review of New Methods for Assessing Food and Energy Intake

**DOI:** 10.3390/nu10081064

**Published:** 2018-08-10

**Authors:** M. Carolina Archundia Herrera, Catherine B. Chan

**Affiliations:** 1 Department of Agriculture, Food and Nutritional Science, Alberta Diabetes Institute, University of Alberta, 6-002 Li Ka Shing Centre for Health Innovation Research, Edmonton, AB T6G 2E1, Canada; archundi@ualberta.ca; 2 Department of Physiology, Alberta Diabetes Institute, University of Alberta, 6-002 Li Ka Shing Centre for Health Innovation Research, Edmonton, AB T6G 2E1, Canada

**Keywords:** dietary assessment, energy intake, validity, reliability

## Abstract

Dietary self-report instruments are essential to nutritional analysis in dietetics practice and their use in research settings has facilitated numerous important discoveries related to nutrition, health and chronic diseases. An important example is obesity, for which measuring changes in energy intake is critical for assessing efficacy of dietary interventions. However, current methods, including counting calories, estimating portion size and using food labels to estimate human energy intake have considerable constraints; consequently, research on new methodologies/technologies has been encouraged to mitigate the present weaknesses. The use of technologies has prompted innovation in dietary analysis. In this review, the strengths and limitations of new approaches have been analyzed based on ease of use, practical limitations, and statistical evaluation of reliability and validity. Their utility is discussed through the lens of the 4Ms of Obesity Assessment and Management, which has been used to evaluate root causes of obesity and help select treatment options.

## 1. Introduction

On a global scale, life expectancy has increased steadily for the past 35 years; however, in association with the global rise of obesity, the number of deaths from most non-communicable causes like diabetes mellitus rose by 32.1%, increasing the burden on health systems [1]. During the past two decades, different Intensive Lifestyle Intervention programs have consistently shown that modest but clinically significant weight loss of 5% in individuals with overweight, obesity or diabetes can yield a variety of health, disease prevention and treatment benefits [2]. Prescription of a hypocaloric diet (500–750 calories less than baseline), increased physical activity (90–175 min/week) and long term behavior change, are common techniques use in Intensive Lifestyle Intervention [3,4,5,6], which also have been described previously as individual techniques for weight control [7,8,9].

Assessment of dietary and/or energy intake (EI) is crucial to understand the impact of clinical trials on the management of obesity and its comorbidities [2,10]. To date, food records (FR), food frequency questionnaires (FFQ), and 24-h recalls (24HR) are the most common methods used to assess dietary and EI during treatment and follow-up [11]. These self-reported data methodologies have provided valuable information to use as a base to develop public health policy, comprehend and identify consumption of different food groups, understand relationship with diseases and determine eating patterns associated with weight loss, information that until recently could not be obtained in any other way [10].

However, a major challenge of these methods is that they rely on self-reported data. Human memory is not 100% accurate in recalling past behavior, consequently these measurements do not directly or objectively measure dietary intake or EI and do not comply with the standards of scientific methodology [12,13]. One issue is that the actual process of doing food records can lead individuals to change their food behavior patterns and therefore, misreport information resulting in an inaccurate report of foods, nutrients and energy consumed [14]. Using data from The National Health and Nutrition Examination Survey (NHANES) 2003–2012, researchers analyzed the prevalence of under and over-reporting of EI, finding that in the US adult population (≥20 years) 25.1% misreported EI [15], results consistent with European countries where prevalence of under-reporting ranges from 20% to a high of 45% [16,17,18,19] with a predominance of obese populations under-reporting. Part of the limitation of behavior modification presented in food records can be overcome though the use of 24HR, since they can be unannounced so that the diet is not changed; however, estimation of the usual diet is weakened by recall bias (food omission or forgetfulness, erroneous estimation of portion size) [20]. In addition to recall bias, these methods impart a substantial researcher/individual burden and high cost of administration [21]. These methodologies used for dietary assessment have been severely criticized to the point of calling the resultant data “pseudoscientific and inadmissible in scientific research”, and what “constitutes the single greatest impediment to actual scientific progress in the fields of obesity and nutrition research” [12].

Thus, the accuracy of dietary assessments or modifications in dietary or EI is full of challenges and the development of new technologies to try to overcome current limitations has been encouraged [21]. The objective of this review is to present the strengths and weaknesses of innovative new tools or methodologies that could replace, improve or complement current self-report dietary assessment instruments.

## 2. Materials and Methods

### 2.1. Search for Innovative Food and EI Assessment Tools and Methodologies

**Figure 1:**
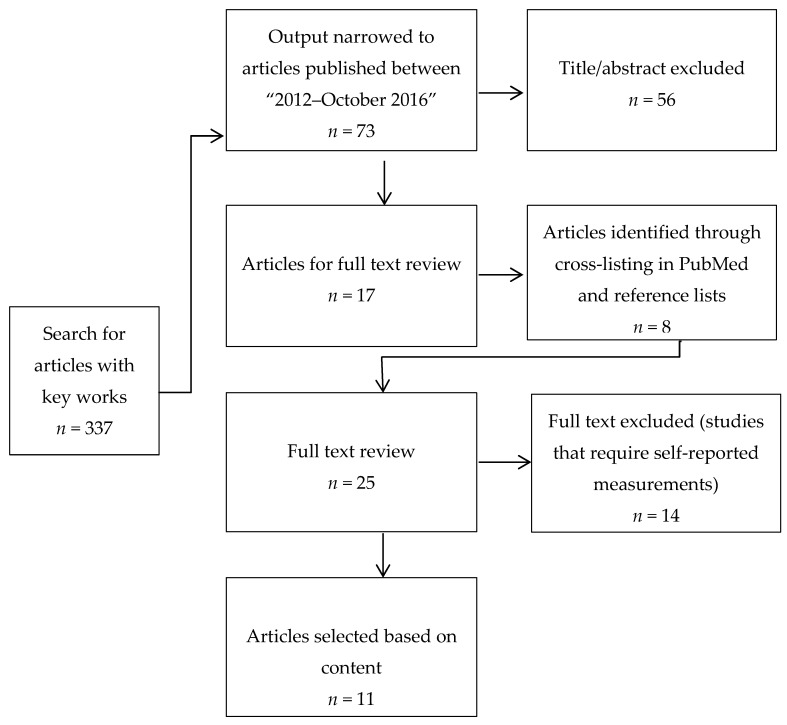
Flow diagram of the articles selection process and exclusion reasons.

Medline, CINAHL and PsychINFO were searched for English-language articles, using the following keywords separately or in combination: diet, diet records, dietary intake, energy intake, innovate *, meals, measurement, metabolism, method, models, new, nutrition assessment, optimiz *, recent, self-report, technolog *, test reliability, test validity, trend, validation studies. The search resulted in 337 articles (Figure 1). The output was then narrowed by imposing search criteria of “2012–October 2016” and “Adults”. This search resulted in 73 articles. These article titles and abstracts were screened by one author (MCAH) to determine if they fulfilled the eligibility criteria. The articles included had to describe or validate a new method, or use new technology tools that could capture food or EI. The methodologies or tools were assessed to determine their benefits and limitations as well as their reliability or validity. Studies using text messaging or mobile phone applications that required manual introduction of information were not considered because using this type of technology imposes the same limitations as the current methods, requiring a self-reported measurement with a burdensome and impractical framework for the subject.

Of the 73 articles uncovered with this search, 17 were considered potentially eligible. These articles were cross-listed in PubMed for articles related to the topic, which identified 8 other articles. Reference lists of relevant articles were also hand-searched but no other relevant articles were found. For these 25 articles, review of the full text was used to identify those meeting the criteria (*n* = 11).

### 2.2. Evaluation

A relative evaluation of the innovative technology tools and methodologies was carried out. They were assessed to ensure that the main weaknesses of present methodologies: recall biases, measurement discrepancies, lack of scientific rigor, were being acknowledged.

When developing tools to collect dietary information, specific statistical methods must be used to evaluate their reliability and validity in order to test the accuracy of the method and avoid bias [22]. Thus, utilization of these recommended statistical methods to assess the different tools and methodologies was noted when drafting this manuscript. *Reliability* refers to “the consistency of a measuring instrument” [22], in different situations; inter-rater, test-retest, inter-method and internal consistency. *Validity* refers to “how close the tool can measure the actual (true) value”; in this case, a measure of true EI when compared to the gold standard [22].

Benefits and limitations: When describing benefits and limitations of each tool/methodology, the focus was on the following criteria: Easy to administer—Referring to reducing participant burden. Current methods rely on information reported by the subjects recalling what they ate for the past week/month/year, or keeping a diary for various days. This decreases the quality of the reports, and the process itself can make subjects change their eating habits [23]. Easy to score—Observed and weighed-food records [24], doubly labelled water (DLW) [25], and FFQ [11], are some of the methods currently used to determine/estimate EI and/or eating behavior. These methods are expensive and time-consuming, making them less feasible to use and hard to score. New methodologies should minimize practitioners’ or researchers’ burden and expense. Capture change over more than one day—Because assessment of day-to-day variability in food intake is an important limitation of current methodologies. New methodology should overcome these limitations and be able to capture fluctuations in habitual energy and nutrient intake on free-living subjects.

## 3. Results

Table 1 summarizes the studies that were included in the review. Five described different types of monitors and sensors; five described camera-scan-sensor-based technologies; and one described a mathematical method. Details of the statistical methods used to assess validity and reliability are noted in Table 2.

### 3.1. Food/Energy Intake Monitoring Devices and Tools

Use of body sensors as a direct measurement of human eating behavior is quite recent. Body monitors and sensors have been developed with the hope of improving and facilitating measurement of daily food and EI [26,27].

#### 3.1.1. Automated Wrist Motion Tracking

The Automated Wrist Motion Tracking, also called a “bite counter” is worn like a watch and automatically tracks wrist motion for monitoring eating in humans [28]. Reliability was tested in both controlled meal and semi-controlled settings. The sensitivity was >85% in both settings. Bites measured by the device were >80% detected compared with bites counted by direct observation. The equations used to measure sensitivity and performance are reported in Table 2 [28]. A third experiment in free-living situations was performed to examine the correlation between bites detected and EI, with *r* = 0.6. This experiment was only exploratory and was done to seek any possible relationships between these factors for further research [28].

Use of this device resulted in improved accuracy of measuring EI in free-living situations compared with 24HR and FFQ, which typically under-report EI in men by 16–20% and 31–36%, and in women by 16–20% and 34–38% respectively [29]. Participant burden was minimal because the user only needed to turn it on and off before eating, and thereafter, bites were registered automatically by the device; thus, researcher and administrative costs are ameliorated since no food weight or labour-intensive laboratory techniques are needed [28,30,31]. Forgetting to use the device, accuracy in different social settings and loss of data when both hands are used to eat are present limitations that the bite counter tool needs to address. Importantly this device’s main benefit is its use as a food intake-monitoring and ingestive behavior tracking system in a real-world setting to improve users, researches and HCP understanding of food intake behaviors. Furthermore, lessen the burden of manual measurements; however, no input regarding the type or quality of the food consumed is tracked.

#### 3.1.2. The Bite-Based Model of Kilocalorie Intake

The bite-counter described above was used for the development of a kilocalorie per bite equation (using bite counts, individual demographic and physical characteristics) that allowed EI to be estimated. The relationship obtained was estimated as kilocalories per bite = −0.128 age + 6.167 sex (female = 0) + 0.034 height + 0.035 weight − 12.012 WHR + 22.294; where WHR = waist-to-hip ratio [30]. The feasibility of using the formula was then systematically evaluated. Two trials were run using a train and test paradigm, in which the training group was used to develop the model and the test group was used to determine reliability of the regression model.

When comparing the reliability of the formula-predicted EI values to the staff-observed values within the test group the Pearson correlation was *r* = 0.374. For the reliability of the model between training and test groups, the difference in r^2^ (which is called the shrinkage value) was 1.4% [30]. To assess validity, researchers assessed participants’ estimation error of EI compared with the equation. The bite-based equation method was more effective at estimating EI than the best human estimation [30].

The bite counter along with the bite-based method formula can provide individuals with a EI estimation that is more accurate than an individual’s estimation even when EI information is available, which potentially could help improve their adherence to recommended dietary changes. The bite counter also has the benefit of being a non-invasive device, which allows tracking of free-living situations for research and also has the potential to improve the understanding of food ingestion patterns including snacking, night eating, and weekend overeating, as pointed out by Fontana 2014 [31] as one of the benefits of food intake monitoring devices and tools. However, reliability was relatively low and internal and external validity of the method needs to be further elucidated. This tool is for monitoring EI purposes only and does not provide information or feedback on diet quality.

#### 3.1.3. The Automatic Ingestion Monitor (AIM)

The Automatic Ingestion Monitor (AIM) integrated hand gestures, jaw motions and accelerometer sensors to detect food intake in free-living individuals [31]. It was designed for objective 24-h monitoring of food intake in free-living conditions without depending on any input from the subjects. The monitor was 90% accurate in its ability to detect specific food intake epochs in free-living individuals compared with self-reported signal (push-button) indicating food intake events, and self-reported food journals [31].

When developing and validating this device, the data were obtained from monitoring free-living situations that included a wide variety of foods and activities, increasing its feasibility for everyday use and research purposes. Its use could provide insight into overall eating behavior patterns where participants burden is minimal. Nonetheless, the use of self-report as the gold-standard method, rather than direct observation, prompts caution regarding reliability. Furthermore, subject compliance with and acceptability of wearing the AIM needs to be established [31]. Insight obtain from this and previous studies [40] encourage further research to build mathematical models to obtain estimated EI using individualized models on counts of chews and swallows (CCS) [32].

#### 3.1.4. Intelligent Food-Intake Monitor

The intelligent food-intake monitor integrates multi-sensor monitors to track chewing speed, and images of the type and amount of food consumed, giving an overall understanding of eating behavior characteristics [33]. The tool was tested for its ability to correctly detect the proportion of food consumed in real life scenarios but results were not reported [33].

The development of the device took into consideration the general process and pattern of food-intake activities to directly target their process (food ingestion, chewing and swallowing). The experiments were conducted in a real-world setting to increase the feasibility of being used in such settings. Valuable information involving eating behavior can be obtained from the use of this device because it doesn´t assume that the food on the plate is consumed, thus providing a more reliable measure than capturing images of food alone to assess food consumption, thanks to the integration of chewing and swallowing detection in the process. Further research needs to be conducted to increase participants’ comfort levels when using the device to ensure compliance with its use for longer periods. Even though a high level of correlation is reported between ground truth and auditory and vision predictors, no *r*-values were given and no strong statistical bases were presented. Participant characteristics were not supplied [33], leaving to speculation the age range that could benefit from using this monitor, and whether it would be feasibility to use in older adults, youth and children.

### 3.2. Camera-Scan-Sensor Based Technologies or Food/Energy Intake Assessment Tools

Sixty-four percent of the American population own a smartphone, a 35% increase since 2011 [41]. Since the use of smartphones is steadily increasing in daily life, mobile phone camera-scan-sensors are being proposed to contribute novel approaches to the measurement of food and EI.

#### 3.2.1. DP + R

Ptomey et al. [34] developed and evaluated a pre-post meal photographic method for assessing EI in overweight and obese individuals in a cafeteria setting. Foods consumed outside this setting were assessed by recall methods.

Nutrition research staff underwent rigorous training for estimating portion size and EI from pre- and post-meal digital photographs and dietary recalls, with inter-rater reliability >95%. DP + R procedure includes taking notes and delineating standard measurements as guidelines for the portion size assessment [34]. The DP + R during ad libitum eating in a cafeteria was compared to measurement of total daily energy expenditure assessed by doubly labelled water (TDEE_DLW_) method [34] with no significant differences found; thus, the method was considered valid.

DP + R method is a reliable and validated method for estimating EI in overweight or obese participants in a cafeteria setting. The main advantage over a food record/recall alone is verification of the written record by the photograph. This method was judged to provide an acceptable level of burden for both participant and research team when compared to previous procedures but a considerable burden is still present for the researcher because of the need to enter nutritional information into a database to quantitate EI [34]. The capacity of DP + R to capture change over time is limited since the procedures are done in cafeteria settings, therefore when the subjects stop attending, the change will not be captured. However, the authors point out the possibility of modifying the DP + R method to use in conjunction with smartphone photos to make the method portable [34].

#### 3.2.2. Remote Food Photography Method (RFPM)

Participants send images taken on their smartphone wirelessly [35] to a Food Photography Application© [42], which is linked to the Food and Nutrient Database for Dietary Studies 3.0 [43]. Trained raters use the application to oversee the semi-automated process of food and nutrient intake estimation [35]. In this trial, some participants received prompts to use the Application customized to their specific meal times, or generic prompts in the morning, at noon and in the late afternoon.

Analyses were run to evaluate any significant differences between the RFPM and DLW estimation of EI, and if they were influenced by the EI consumed; no significant differences were found when participants received customized reminder messages but device reliability was decreased when participants received generic prompts [35]. The RFPM and DLW were used to measure EI in free-living individuals during a 6-day period. The error between methods (EI estimated with the RFPM minus EI measured with DLW) was calculated and was smaller in the participants receiving customized prompts [35].

The underestimation of EI by RFPM improves drastically compared to self-report methods, particularly when accompanied by customized prompts, allowing monitoring of habitual EI in free-living individuals. The method also offers the opportunity to detect missing data (due to technical problems or no compliance) promptly, and take pertinent action (contact participant) to improve data quality and compliance, thereby reducing recall bias [44]. The ability of RFPM to provide users feedback about their behavior is another benefit worth mentioning. In general, the user burden is keep to a minimal and 82% of users rated overall satisfaction 5 or higher (based on a six-point scale) [35]. However, since the method is only semi-automated, it remains expensive to analyze. The RFPM has also been used to estimate EI in children in both research and free-living settings [35,42,44].

#### 3.2.3. Real-Time Food Recognition System

The user points the smartphone camera at the food plate for the food recognition process. After selection of the food from a database and indication of its approximate volume, the calorie and nutrition values are displayed [36]. The real-time recognition of foods was approximately 80% correct.

This system utilizes a real-time image recognition system, and the processing time only takes 0.065 s once the user enters the input. A fully automated interface with a food database completes its system. Evaluation of its usability was carried out, where adjustment of the bounding boxes on the different food items wasn’t as positively rated (2.4 out of 5) as for the item recognition itself, which was done automatically without additional user input, obtaining an average score of 4.2 out of 5 [36]. However, this tool hasn’t been validated and has a limited number of food categories and it does not specify the database used for the nutrition information.

#### 3.2.4. “Snap-n-Eat”

A “snap-shot” (photograph) of participants’ plate is captured. The analytical system is based on predefined EI and nutritional density for each food category. Depth images are used to estimate the portion size of the food and the EI and nutritional content are displayed on the user’s screen in ~4 s [37]. A classification accuracy (the percentage of the test images of each category correctly classified) of 85% was obtained for 15 different food categories.

Snap-n-Eat presents a food recognition system for which users only need to take a snapshot of their food in order for the system to estimate its EI and nutritional content allowing participants to track their daily food intakes helping to understand their eating habits in a cost and time effective manner. However, in order to be a feasible tool, a scale-up to hundreds of food items and a validation process is needed [37].

#### 3.2.5. GoCARB

The user photographs their food from at least two angles. The food items are segmented and recognised and their carbohydrate content is estimated based on the nutritional information of the USDA Nutrient Database for Standard Reference [38]. GoCARB’s portion sizing and individual food item recognition accuracy ratings were 75% and 85%, respectively [38]. To validate the device, adult participants with type 1 diabetes were asked to calculate the carbohydrate content of the meals by themselves and subsequently with the help of the GoCARB. The error using GoCARB error was approximately half of that without any aid [38].

The application is overall better than participants at estimating carbohydrate content of meals. In the GoCARB app the carbohydrate content estimation is done automatically so the burden on researchers and participants is minimal; thus, 90% (17/19) qualify the tool as easy to use and would like to use the application on a regular basis. These measurements were done in a clinical setting that may not represent real-life situations where the meals may have more complex composition than the test meals. The overall nutrient content of the meal is not analyzed, and for individuals with diabetes it is important to consider the influence of the overall meal in determining their postprandial glycemia [38].

### 3.3. Mathematical Algorithm

A totally different, novel approach to assess EI is through mathematical algorithms.

#### Mathematical Method

Sanghvi’s group [39] validated a mathematical formula originating from the National Institute of Diabetes and Digestive and Kidney Diseases (NIDDK) [45], to measure long-term changes in free-living EI of humans by using repeated DLW/DXA measurements collected over 2 years in 140 free-living subjects from the Comprehensive Assessment of Long-term Effects of Reducing Intake of Energy (CALERIE) study [46]. The formula inputs were baseline demographic (age, sex, height) and repeated body weight data, which was used to obtain the change in body weight over time and the rolling average.

Measured body weight and EI changes for the participants were documented over 2 years at 4 different time intervals. During the course of the study, the test-retest reliability was obtained by comparing the gold standard with the mathematical model, differing by only 40 kcal/day [39]. The change in EI values calculated by the mathematical method was compared (paired, 2-sided *t*-test) to the gold standards DLW/DXA and found to be similar [39].

In order for the formula to measure long-term changes in free-living EI, easily acquired initial information regarding age, sex, height and physical activity are required. However, baseline DLW measurements are also needed to establish energy requirements if one wishes to know absolute EI as well as changes in EI over time, limiting its use to researchers with the ability to obtain this parameter [39]. If all the mathematical parameters are available, the formula is an easy-to-score tool that captures changes over more than one day; however, the model might require adjustments for use in children or older adults. In addition, specific nutrient/food intake information is not known, therefore without co-administration of a diet record or FFQ it would not be possible to obtain this information [39]. Important limitations must be considered. The study was conducted on normal weight individuals and validation in individuals with obesity was not demonstrated even though the authors were confident the model could be used on this population because the model was built to measure changes in metabolism and body composition.

## 4. Discussion

From a research perspective, the first and foremost goal of evaluating food and EI is to be able to increase our understanding of diet-disease associations. Validated and reliable measures of food and EI are crucial to understand their relationship with health, especially with the overwhelming increase in obesity prevalence [1]. Individuals with obesity present different problems ranging from the physiological to the psychological aspects, which represent barriers to their treatment. The 4Ms of Obesity Assessment and Management (Mental, Mechanical, Metabolic and Monetary) has been proposed as a framework to help identify the root cause and help obesity treatment [47].

The methodologies/tools presented in this review have the potential to aid in the understanding and treatment of obesity within this framework. This review identified 3 main new modalities for estimating food and EI. These include devices that monitor intake through sensors that detect movement of the arm and/or jaw, counts of chews and swallows, smartphone-based photographic methods linked to food databases and a mathematical formula.

In order to come to a consensus of which methodology/technology would be the most highly recommended it is important not to lose sight of why EI is being assessed or monitored. The overall objective should guide opting for one or the other.

In the context of the 4Ms of Obesity Assessment and Management, if the individual being treated is believed to have psychological (Mental) issues influencing their eating behavior, then the main objective is to understand their eating behaviors or food intake patterns in order to detect and/or modify eating habits. Food intake-monitoring devices and tools (Bite Counter, AIM, Intelligent food-intake monitor) would be recommended in this context because they could provide useful insight regarding food intake behaviors (e.g., timing and size of meals). In general, food intake-monitoring devices and tools can count the number of bites an individual takes, track the approximate EI and monitor episodes of food intake. Several benefits to the understanding of food intake behaviors may accrue from these methods. These devices could fill a gap in providing timely monitoring and feedback to individuals wishing to change eating habits, similar to the way the use of pedometers and/or accelerometers has been validated to promote and assess physical activity [48], by establishing and monitoring personal goals achievement, a behavior that according to Social Cognitive Theory, is an effective behavior change strategy [49] aiding behavioral change. Therefore, these tools could be used for monitoring, controlling and correcting eating behaviors and portion size in obese or overweight individuals as well as for chronic disease management. However, their effectiveness in eliciting behavior change has yet to be documented. Future work includes the possible addition of a vibrotactile alarm, similar to the technology used on intelligent watches or pedometers so that subjects can self-adjust their eating behavior based on the estimated EI per bite [28]. Moreover, the commercial cost of these devices has not been established since they are still on the development phase and have not gone further to establish a market cost. Further, wearing some devices may be more acceptable to participants than others.

On the other hand, if the aspect of obesity treatment is within the Metabolic category of the 4Ms, as in the case of individuals with T2D or hypertension, then the intent would shift the focus to understanding specific macro/micronutrient intakes (sugars, salt, fats). Similarly, within the Mechanical category (such as osteoarthritis), weight loss could be desired to reduce pain. For both approaches, camera-scan-sensor (Snap-n-Eat, GoCARB) could be useful. RFPM could be applied in a hospital setting where monitoring individuals’ nutrition intake is essential but difficult to do on a routine basis. Registered dietitians and nurses could use this tool to oversee adequate food intake essential for hospitalized individuals’ wellbeing. If the overall objective is a focus on measures of long-term changes in EI in free-living individuals undergoing a research or lifestyle intervention, the mathematical method would highly be recommended since its accuracy lies within 40 kcal/day of mean difference with the gold standard, as long as the initial DLW measurement is possible to obtain, which could be a potential limitation.

Overall, the studies included in this review presented new devices designed to improve how EI is measured, analyzed and registered. However, the devices and methods have usually undergone pilot testing in small numbers of participants and various limitations elicit caution. Food intake-monitoring tools have limited ability to assess day-to-day variability in food intake [28,30,31]. They do not take into account the type of food consumed, its EI density nor its consistency; therefore, no information about the macro/micronutrient is obtained, resulting in a inability to capture change in type of food or nutritional intake over time. As mentioned previously, a current limitation with present methodologies used to assess food intake or EI is individual reactivity causing changes in food behavior patterns, thereby resulting in inaccurate reporting. None of the present studies addressed these issues, therefore the question arises: could bias play a role in the use of these devices? That is, would peoples’ consumption of food intake be modified by simply wearing these tools? And if so, what would be the differences compared with current methodologies? Certainly, more accurate data of consumption patterns seems possible, but to date none of the devices has gone beyond pilot testing nor addressing potential bias. To our knowledge, the application of these methodologies to clinical settings or outside of the original developers’ laboratories has not been reported.

Regarding smartphone-based apps, additional limitations applying to one or more include participants forgetting to take the photographs, or not having the smartphone with them [35]. In general, two major limitations need to be addressed with camera-scan-sensor methods. First, they cannot quantify all food ingredients or beverages. These tools only work with the food items in the database of each individual tool, and their validity is also dependent on the food nutrient value on which the databases are built. However, with current food record/recall databases, there are acknowledged differences between what a person consumes and what the database contains [38]; even the Canadian Nutrient File or the USDA database cannot keep up with constantly evolving food possibilities. The use of these technologies is not advanced enough to correctly and accurately estimate 100% of food intake since the best achieved accuracy was 85% based on a small number of foods [37]. Second, they cannot judge quality since a photograph doesn’t convey information about ingredients that are hidden or blended [35]. Nevertheless, these methods show improvement in estimating, on average, the nutrient content of meals more accurately, easier and faster than individuals’ self-report measures (24HR, FFQ, etc.) but caution must be taken when using and analyzing these methods. Bearing in mind the strong link between food intake and health, continuing to document the improved validity and reliability of the food item recognition and nutritional information provided by these tools would undoubtedly lead to better outcome measurement in the fields of obesity and nutrition. However, the feasibility creating comprehensive databases for food recognition is problematic in an environment of incessantly increasing food possibilities. On the other hand, the ubiquity of smartphone ownership means that affordability and acceptability are of less concern with the main investment being the data processing.

## 5. Conclusions

In conclusion, these innovative dietary assessment tools are able to record food/energy intake more accurately than participants’ estimates and are an improvement on important weaknesses of conventional methods (paper-based records/recalls), particularly regarding the burden of recording by participants and collecting/administering and evaluating/scoring the information by researchers. However, caution is needed when using them since they are still being refined. Future work should look at combining body monitor sensors and camera-scan-sensors to work together in order to counter their strengths and weaknesses. This work should eventually progress outside of research settings and promote the collaboration of dietitians with engineers to co-develop the design, development, evaluation and implementation of these new tools, since this would likely increase their effectiveness, acceptability and validity. Lastly, this research field should take into consideration changing formats of national nutrition recommendations, such as the 2014 Brazilian dietary guidelines, 2015 Dietary Guidelines for Americans and the American Heart Association, which are shifting the focus from single nutrients or kilocalorie counting into healthy eating patterns [50,51,52]. Therefore, future development should aim at being able to detect overall eating patterns.

## Figures and Tables

**Table 1:** Summary of New Methods for Assessing Food and Energy Intake.

Reference	Objective	Brief Description	Key Findings
[28]	Evaluate a new method of automated dietary intake monitoring.	The “Bite Counter” device was worn like a watch. Before eating, the user pressed a button to turn it on (and off afterwards). While operating, the device used a micro-electro-mechanical gyroscope to track wrist motion, automatically detecting when the user had taken a bite.	The method worked across a reasonably large number of subjects, and variety of foods, and there was modest correlation with EI on a per-meal level.
[30]	Evaluate accuracy of an individualized bite-based equation of kilocalorie intake compared to participant estimates of kilocalorie intake.	Subjects’ real kilocalorie intake was compared to predicted kilocalories estimated by: (a) the bite-based equation of kilocalorie intake, (b) participants’ kilocalorie estimate when provided with kilocalorie information of the foods eaten, (c) participants’ kilocalorie estimate without kilocalorie information.	The bite-based equation measure of kilocalorie intake outperformed human estimates with and without menu kilocalorie information.
[31]	Evaluate the: Automatic Ingestion Monitor (AIM) for objective detection of food intake in free-living individuals.	The AIM integrated three sensor modalities and a pattern recognition method for subject-independent food intake recognition.	The AIM can detected food intake with an average accuracy of 89.8% suggesting that it can be used to monitor eating behavior in free-living individuals. AIM could be used as a behavioral modification tool.
[32]	Estimate EI using individualized models based on Counts of Chews and Swallows (CCS).	EI was estimated by the CCS mathematical model and compared to the weighed food records, diet diaries and photographic food records methods.	Mathematical models based on the CCS could be potentially used to estimate EI.
[33]	Present an intelligent food-intake monitoring system that can automatically detect eating activities	The multi-sensor monitor detected chewing activity via its integrated ear-microphone, consequently the camera was activated, snapshots for food detection were taken.	The high correlation rates reported (*r* not shown) suggested the usefulness of the proposed method to provide with an overall understanding of eating behavior characteristics (speed, type and amount of food consumed).
[34]	To compare mean EI of overweight and obese young adults assessed by a Digital Photography + Recall method (DP + R), to the mean total daily energy expenditure assessed by TDEE_DLW_.	Two digital still photographs (90° and 45° angle) were taken by a digital camera approximately 30 inches above the tray. Notes were placed on the tray to identify types of beverages and standard measures were included to guide the assessment of portion size. The type and amounts of food and beverages consumed and results from recalls were entered into the Nutrition Data System for Research to quantification for EI. TDEE_DLW_ was assessed in all participants to compare mean daily EI.	The mean EI estimated by DP + R and TDEE_DLW_ was not significantly different (*p* = 0.42). On average, DP + R overestimated EI compared to TDEE_DLW_ by 6.8 ± 28%.
[35]	To validate the Remote Food Photography Method (RFPM)	Developed for automating dietary assessment. Participants include a reference card placed next to the food plate as well as labels of not easily recognizable foods for the portion size estimation to take place. A barcode reader phone app and a voice message option are innovations included to facilitate identification of foods. Participant received feedback about their food intake behavior and recommendations to achieve weight goals. To maximize and promote usage of RFPM in free-living conditions, ecological momentary assessment (EMA) methods were adopted, which involves sending small reminders or prompts to the user via email or text message. EMA was tested by comparing two groups; the standard prompts (2 or 3 prompts a day send to their smartphones around meal time) versus customized prompts (3 to 4 personalized prompts, send at participants’ specific meal time).	The RFPM and DLW did not differ significantly at estimating free-living EI (−152 ± 694 kcal/day, *p* = 0.16) nor did it differ when estimating energy and macronutrient intake.
[36]	To evaluate a mobile food recognition system which estimates calorie and nutritional components of food intake.	(1) User pointed the smartphone camera to the food (2) Drew bounding boxes to delimit food regions (3) Food item recognition started within the indicated bounding boxes. To recognize them more accurately each food item region is segmented by GrubCut. The recognition process results in a display of the top 5 food item candidates. The user selects the most accurate candidate and indicates the relative approximate volume of the food.	A 79.2% classification rate was achieved. The recognition processing time was only 0.065 s.
[37]	To present Snap-n-Eat, a mobile food recognition system.	The user took a photo of the plate. The system detects the salient regions corresponding to the food items. Hierarchical segmentation was performed to segment the images into regions. The system estimated the portion size of the food and uses it to determine the EI and nutritional content.	The Snap-n-Eat application achieved a 85% accuracy when detecting 15 different categories of food items. Snap-n-eat recognized foods presented on a plate and estimated their caloric EI and nutrition content automatically without any user intervention.
[38]	To assess the accuracy of the GoCARB prototype when used by individuals with type 1 diabetes and to compare it to their own performance in carbohydrate counting.	The user placed a reference card next to the dish and took two images using a mobile phone. A series of computer vision modules detected the plate and automatically segmented and recognized the different food items, while their 3D shape was reconstructed. The carbohydrate content was calculated by combining the volume of each food item with the nutritional information provided by the USDA Food and Nutrient Database.	GoCARB was more accurate at estimating carbohydrates content than individuals with type 1 diabetes. The mean absolute estimation error while using GoCARB was reduced by more than 50% than without using GoCARB.
[39]	To validate a mathematical method to measure long-term changes in free-living EI	DLW was used to assess Energy Expenditure (EE) at months 6, 12, 18, and 24. DXA and body weight measurements were taken twice at baseline, twice at month 6, and once at months 12, 18, and 24. Body weight measurements were taken at months 1, 3, 6, 9, 12, 18, and 24 in the CALERIE study. Then, they compared the ΔEI values calculated by using DLW/DXA with those obtained by using the mathematical model	The mean (95% CI) ΔEI values calculated by the model were within 40 kcal/day of the DLW/DXA method and were not significantly different throughout the 4 times segment (*p* = 0.14, *p* = 0.34, *p* = 0.32, *p* = 0.11). Most of the model-calculated ΔEI values were within 132 kcal/day of the DLW/DXA method.

**Table 2:** Summary of the Reliability and Validity of New Methods for Assessing Food and Energy Intake.

Reference	Name of Tool	What Is Measured	Reliability		Validity	
			Statistical Method Used	Result	Statistical Method Used	Result
[28]	Automated Wrist Motion Tracking	EI	Sensitivity (true detection rate) = (total true detection)/(total true detection + total undetected bites); Positive Predicted Value (PPV) = (total true detection)/(total true detection + total false detection); compared recorded bites with direct observation.	Control setting: Sensitivity = 94% PPV = 80% Semi-controlled setting: Sensitivity = 86% PPV = 81%	Pearson correlation of EI estimated by device vs. direct observation (*r*)	R = 0.6
[30]	The bite-based model of kilocalorie intake	EI	Pearson’s correlation of device compared with direct observation; shrinkage value	R = 0.374 Shrinkage value (difference in R^2^) = 0.014	Independent *t* test Paired sample *t* test	Mean estimation error kilocalorie information group: −185 ± 501 kcal; Mean estimation error no kilocalorie information group: −349 ± 748 kcal (*p* < 0.05); Best human-based estimation (kilocalorie information group) mean estimation error: −257 ± 790 kcal; Bite-based method (predicted formula) mean estimation error: 71 ± 562 kcal; (*p* < 0.001).
[31]	Automatic Ingestion Monitor (AIM)	EI	N/A	N/A	Accuracy = average between precision (P) and recall (R).	Accuracy of food ingestion = 89.9%, range from 75.82–97.7%.
[32]	Counts of Chews and Swallows Model	EI	A 3-fold cross validation technique, one sided Wilcoxon-Mann-Witney, Bland-Altman analysis and *t*-Test analysis.	Reporting error for the CCS model was lower than that of the diet diary (*p* < 0.01). The model underestimated EI. Energy intake estimation had the lowest bias.	A 3-fold cross validation technique, one-sided Wilcoxon-Mann-Witney, Bland-Altman analysis and *t*-Test analysis.	No statistical differences were found between the CCS model and either diet diary or photographic records.
[33]	Intelligent food-intake monitor	Food intake	Correlation: Proportion of food consumed from sound (auditory based) and image sequence (vision based) compared to the ground truth: proportion of food consumed.	Data not shown	N/A	N/A
[34]	DP + R	EI	Inter-rater reliability coefficients	Error rate ≤5%, Recall assessments ≥0.95	Dependent *t*-test comparing device to DLW method; Bland-Altman plots; Limits of agreement	Differences between methods in the total sample was not significantly different (DP + R = 2912 ± 661 kcal/day; TDEE_DLW_ = 2849 ± 748 kcal/day, *p* = 0.42); DP + R was found to overestimate EI compared to TDEE_DLW_ by 63 ± 750 kcal/day (6.8 ± 28%; limits of agreement: −1437, 1564 kcal/day). The Bland-Altman plot indicated no proportional bias variation as a function of the level of EI in the total sample (R = −0.13, *p* = 0.21).
[35]	RFPM	EI	Bland & Altman analysis	Significant difference: *p* < 0.0001 between the RFPM and DLW in the standard prompt group. No significant difference in the customized group: *p* = 0.22. The level of bias in both groups was not influenced by the amount of EI (Adj. R^2^= −0.03, *p* = 0.55; Adj. R^2^ = −0.08, *p* = 0.78)	Independent sample *t*-test for error between methods = EI estimated with the RFPM-EI measured with DLW	Significant smaller underestimation in the customized group (270 ± 748 kcal/day or 8.8 ± 29.8%) when compared to the standard prompt group (895 ± 770 kcal/day or 34.3 ± 28.2%), *t* (33) = −2.35, *p* < 0.05 with RFPM.
[36]	Real-time Food Recognition System	EI	Test-retest reliability	79.2% classification rate	N/A	N/A
[37]	Snap-n-Eat	Energy/dietary intake	Test-retest reliability	Classification accuracy (% of correctly classified images categories) = 85%	N/A	N/A
[38]	GoCARB	Carb EI	Comparison to actual foods/database	Automatic segmentation (portion size) = 75.4% (86/114); Food item recognition = 85.1% (291/342)	Mean absolute error; Relative error	Mean absolute error = 27.89 (SD 38.20) and 12.28 (SD 9.56) grams of carbohydrates; Mean relative error = 54.8% (SD 72.3%) and 26.2% (SD 18.7%). A significant error between estimations was found (*p* = 0.001). In general, 60.5% (69/114) of the participants underestimated carbohydrate content.
[39]	Mathematical method	Change in EI	Test-retest reliability; Mean difference	40 kcal/day of mean difference between the gold standard and the mathematical model; No significant difference between the methods for any of the time segments was found (weeks 0–26: *p* = 0.14; weeks 26–52: *p* = 0.34; weeks 52–78: *p* = 0.32; weeks 78–104: *p* = 0.11).	Paired, 2-sided t test; Pearson correlation (*r*) Spearman’s corrected (*rs*)	Change in EI values calculated by the mathematical method or the gold standard DLW/DXA weren’t significantly different; The mathematical model had an accuracy within 132kcal/day for predicting changes in EI; The magnitude of correlation of the change in EI values between models were correlated (weeks 0–26: *r* = 0.57 (95% confidence interval 0.45, 0.68); *p* =≤ 0.0001; weeks 78–104: *r* = 0.19 (0, 0.36); *p* = 0.05).

^1^ N/A = Not applicable.
